# Self-Concept and Support Experienced in School as Key Variables for the Motivation of Women Enrolled in STEM Subjects With a Low and Moderate Proportion of Females

**DOI:** 10.3389/fpsyg.2019.01242

**Published:** 2019-06-26

**Authors:** Silke Luttenberger, Manuela Paechter, Bernhard Ertl

**Affiliations:** ^1^Institute in Early Childhood and Primary Teacher Education, University College of Teacher Education Styria, Graz, Austria; ^2^Educational Psychology, Institute of Psychology, University of Graz, Graz, Austria; ^3^Learning and Teaching with Media, Department of Education, Universität der Bundeswehr München, Neubiberg, Germany

**Keywords:** gender, STEM, motivation, academic self-concept, school factors, university students, latent regression analysis

## Abstract

The proportion of women enrolled in STEM courses at university level has remained consistently low for decades. Differences, however, exist between various STEM domains: While engineering and technology appear especially unattractive, subjects such as mathematics, biology, or chemistry have better chances at attracting women. Research has mostly neglected these differences, treating STEM as an overall category. In the light of the differences in the proportions of women enrolled in and dropping out of various STEM subjects, the present study takes a more differentiated look to separately investigate the STEM subjects that have a low or moderate proportion of females. The following study focuses on female university students’ intrinsic and extrinsic motivations in these two groups of STEM subjects, asking to what degree the academic STEM self-concept and support experienced in both school and the family contribute to the motivation to study a STEM topic. Four hundred sixty-nine female students took part in the investigation. Two hundred eighty-four of them were enrolled in STEM subjects with a low proportion of females (STEM-LPF) and 185 in STEM subjects with a moderate proportion of females (STEM-MPF). A comparison of the two samples shows that women in STEM-LPF exceed women in STEM-MPF with regard to their academic STEM self-concept and intrinsic and extrinsic motivations. Different variables contribute to motivation in the two samples. For STEM-LPF, a latent regression analysis found positive relationships between the academic STEM self-concept and both intrinsic and extrinsic motivations, while support experienced in school and from the family was not related to motivation. By contrast, in the STEM-MPF sample, the academic self-concept was not related to motivation. Previous interest in STEM subjects in school contributed positively to present intrinsic and extrinsic motivations. An unexpected result, however, was found concerning activities in school that were designed to promote interest in STEM. Memories of these kinds of activities were negatively related to both intrinsic and extrinsic motivations. These measures might be experienced as intrusive support: attempts to promote STEM sometimes might backfire and achieve the opposite of what was intended.

## Introduction

Not many women pursue a career in the STEM fields (**S**cience, **T**echnology, **E**ngineering, and **M**athematics). This is particularly the case for engineering and technology. The proportion of females in these fields has remained consistently low over the last decades (e.g., Blickenstaff, 2005; Ihsen et al., 2013) – in the EU, it was only 25.7%, with Germany and its 18.5% scoring even lower (Center of Excellence Women and Science, 2014). There is also an underrepresentation of women in mathematics and sciences, although the difference is less severe, with a proportion of 37% in the EU and 35.6% in Germany (Center of Excellence Women and Science, 2014). Altogether, there are differences in the amounts of women in STEM between different EU member states, as well as between particular STEM fields. One reason for this might be that attitudes toward and attributions to subjects and professions vary between different cultural and subject-specific backgrounds (Else-Quest et al., 2010; Nosek and Smyth, 2011). Nosek and Smyth (2011) point out that women in the Western world still hold strong beliefs about STEM being a “male” domain.

And it is not just that women are less likely to enroll in STEM. When enrolled, they are more likely to drop out of their studies. Two variables in particular are seen as decisive for enrollment and dropout rates: STEM self-concept and motivation (Ellis et al., 2016). These will also serve as key variables in the present study, which investigate to what degree personal variables like the academic self-concept, as well as socializing school- and family-related factors contribute to women’s intrinsic and extrinsic motivations in STEM.

In light of the variations in enrollment as well as dropout rates in different STEM subjects (e.g., higher enrollment of women in biology or chemistry than physics or engineering, Watt et al., 2017), it appears inadvisable to treat STEM as a general category. Watt and colleagues argue that it is “imperative to disaggregate discussions of different fields of sciences rather than use an aggregated concept of STEM” (Watt et al., 2017, p. 254). However, in light of an apparently infinite number of STEM majors, it is neither practical nor efficient to separately investigate subjects, which is why this study chooses another approach, classifying STEM majors according to the proportion of women enrolled in them.

The proportion of women in a field is a critical variable. It appears that personal attitudes, assessments, and characteristics are related to enrollment in subjects with a lower or higher proportion of women (Ihsen et al., 2013; Ertl et al., 2014). Gender disparities create a social context in STEM fields that signals to females that they are “minorities” who may not belong there (Murphy et al., 2007) and/or that women will encounter specific obstacles in these subjects. Therefore, the present study investigates the contribution of personal and social variables to motivation in two STEM groups with different proportions of women enrolled in the respective STEM fields.

## Motivation, Self-Concept, and Socializing Family- and School-Related Factors in Stem

Considering impacts on career choice as well as persistence in a study or a course, one group of theoretical models and research studies focuses on motivation as a driving force. From a view point of motivation, intrinsic motivation, interest, enjoyment, the experience of self-determination as well as persistence, and the wish to achieve certain goals are important reasons to pursue a certain career path (e.g., Dickhäuser and Meyer, 2006; Ihsen et al., 2013; Van Soom and Donche, 2014). By contrast, models and research studies within a socio-cultural background focus more strongly on socializing factors and the cultural environment of a person (e.g., Dick and Rallis, 1991; Adya and Kaiser, 2005).

### Motivation

Motivation plays a crucial role when it comes to learning behaviors, career choice, as well as persistence (e.g., Dickhäuser and Meyer, 2006; Ihsen et al., 2013). It describes the combination of a trait-like preference and a positively experienced, situation-specific state when working on a task (Macher et al., 2013). Motivation explains to which degree an individual makes an effort to achieve a particular goal, e.g., a good test score or a degree.

According to self-determination theory (Deci and Ryan, 1991), an individual can be motivated by internal rewards, e.g., enjoyment in doing, exploring, and learning things (intrinsic motivation) or by external rewards, e.g., money or prestige (extrinsic motivation). Intrinsic and extrinsic motivations can be ordered along a continuum. Intrinsic motivation can be described as an experience of competency or autonomy that manifests itself in sustainable efforts over a longer range of time. It is autonomous in the sense that it is experienced as self-determined (Van Soom and Donche, 2014). With regard to extrinsic motivation, self-determination theory distinguishes different forms. It comes from external sources (like external rewards) and is more goal driven and less sustainable. However, extrinsic motivation varies to which degree it is externally triggered; it may have internal sources when personal importance is placed on rewards (e.g., a high salary; Van Soom and Donche, 2014). Extrinsic motivation can serve as an impetus to put effort into learning, e.g., when passing a test is instrumental toward obtaining rewards.

The motivation to enroll in a STEM major and stay on a chosen STEM career path usually results from a combination of both high intrinsic and extrinsic motivations (Aeschlimann et al., 2016), with male STEM students’ motivation mostly exceeding that of female students (Ihsen et al., 2013). With regard to motivation in STEM, mathematics has been identified as a critical filter, which excludes girls and women from joining and/or remaining in a STEM field of study (Else-Quest et al., 2010). Mathematics is regarded as a typically male domain, even though achievements have not differed across genders in many studies (Stevenson and Newman, 1986). Nevertheless, boys and men feel more confident overall when solving mathematical problems and have more positive attitudes toward and a higher motivation in mathematics (Vermeer et al., 2000; Else-Quest et al., 2010; Lazarides and Ittel, 2012).

### Self-Concept

Self-concept is defined as the trait-like knowledge and perception about oneself. It is multidimensional, having both a nonacademic and an academic self-concept. These two overarching self-concepts are made up of more specific ones such as self-concepts in different academic domains (e.g., math, languages; Marsh and Scalas, 2011).

The academic self-concept is formed already in childhood by experiences, the feedback of others, as well as by attributions to a person’s behavior and achievements, and it becomes more stable over time (Luttenberger et al., 2018). Self-concepts in different academic domains comprise an individual’s self-assessments and perceptions of competence (“I am good in science”). These kinds of self-assessments may rely on different frames of reference. An external frame of reference uses comparisons of own achievements to the achievements of peers or to a predetermined performance standard, while an internal frame of reference compares former achievements to present ones (Altermatt et al., 2002; Schunk et al., 2014). Assessments can additionally rely on a general perception of individual abilities without the use of a certain frame of reference (Hoferichter et al., 2018).

Self-concept and achievements are related to each other *via* learning behaviors and effort (Valentine et al., 2004). A higher self-concept results in higher effort and persistence and thus implies higher achievements, while high achievements conversely strengthen the self-concept (Marsh and Scalas, 2011). The overall academic self-concept (how quickly I learn and how well I generally do at school) and the domain-specific self-concept (e.g., how good I am in mathematics) may influence preferences and choices of courses, school types, and even professions.

In STEM subjects, women often have a more negative self-concept than males, even if they in fact have the same grades and achievements (Jacobs et al., 2002; Watt, 2004; Frenzel et al., 2010; Nagy et al., 2010). Girls are more likely to attribute success to external factors and failure to internal factors such as a lack of mathematical ability (Parsons et al., 1984). Because mathematics is a crucial filter for enrollment as well as for remaining in STEM education, a lower math self-concept can be detrimental and may also lower learning motivation (Dresel et al., 2007). Generally, an overly critical math and STEM self-concept is a significant factor impacting why females are less motivated in STEM subjects and why they seldom consider a career in STEM (OECD, 2015). These differences can be partially seen as the results of socialization at home and school because the gender-specific math and STEM self-concepts become increasingly significant following primary school (Senler and Sungur, 2009).

### School and Family as Socializing Factors

Social cognitive theory emphasizes the role of observing and interacting with others for an individual’s personal development. In an ongoing process, individuals learn from significant others (as models), but individuals also vary in what they adopt from significant others and they are of course influenced by the consequences of their behavior and the interactions with others (Bussek and Bandura, 1999). As such significant others, teachers and parents or the closer social context are socializers (Watt et al., 2017), which influence the development of students’ academic self-concept as well as their motivation in a field.

Teachers influence their students’ self-concept and motivation in different ways. Cognitive activation, style of teaching, feedback, as well as teachers’ attributions to achievement are important for the development of students’ motivation in a subject (Lazarides and Ittel, 2012). Teachers can help students overcome gender-specific attribution patterns by, e.g., encouraging girls to attribute success to their own abilities and not external reasons, achieving motivation as a result (Dresel et al., 2007).

In a similar fashion, parents and the closer social context shape both the self-concept and motivation. Parents’ beliefs about their children’s abilities, as well as their feedback and support influence their children’s self-concept and motivation (Tiedemann, 2000; Dresel et al., 2007; Gunderson et al., 2012; Watt et al., 2017). Parents also shape their children’s career decisions by feedback, role modeling (Kessels, 2015), or content-specific support. In this context, students’ interpretations of the social environment are crucial. According to Watt et al. (2017), career choice is more strongly influenced by how children and adolescents perceive parental positions than by what parents themselves report about their support. In a survey on careers in science, female researchers emphasized more than males the impact of their parents, particularly of the father, on their career choice (Sonnert, 2009). Besides parents, the support of peers and friends plays an important role for STEM career decisions (Robnett and Leaper, 2012). Dick and Rallis (1991) postulate in their model on career choice that aptitudes, experiences, socialization factors, and the cultural milieu have an impact on career-specific values, motivation, the self-concept, and career choice as a result.

## Research Questions

As indicated, motivation is a crucial factor for enrollment and persistence in a STEM field. The present study investigates the contributing factors to the academic STEM self-concept and socializing factors (perceived family and school support) on intrinsic and extrinsic motivations in STEM. Taking into consideration the variations between different STEM fields, this study investigates two samples: Women studying STEM subjects with a low (STEM-LPF) and a moderate proportion of females (STEM-MPF). The following research questions will be investigated:

1. To what degree do STEM-LPF students and STEM-MPF students differ with regard to motivation, STEM self-concept, and socializing factors?
2. How do STEM self-concept and socializing factors contribute to motivation of (1) STEM-LPF students and (2) STEM-MPF students?

## Materials and Methods

### Participants

The sample of this study is female German university students. They were surveyed in the context of a larger study in six European countries by the EU research project SESTEM, a project on equality of job opportunities. Students were contacted through university mailing lists and invited to participate in the study. The sample comprises primarily students in undergraduate and Master’s level courses for STEM and STEM teaching.

Following the recommendation by Buchmann et al. (2002) to use a proportion of 30% as a critical threshold value for identifying typically male and more integrated professions, two samples of women were identified: 284 women in STEM-LPF (STEM fields with a low proportion of females, equal to or lower than 30%) and 185 women in STEM-MPF (STEM fields with a moderate proportion of females, higher than 30% but lower than 70%). All participants were enrolled in German universities. Women in the STEM-LPF sample studied the following subjects (ordered according to the number of participants enrolled in the respective fields): mechanical engineering, computer sciences, physics, metal engineering, electrical engineering, civil construction, or other kinds of engineering subjects (including subject combinations). STEM-MPF students studied mathematics, biology, geography, chemistry, STEM teacher education, biotechnology, architecture, or other subjects (ordered according to the number of participants enrolled in the respective fields). Some STEM-MPF students studied a STEM subject plus a non-STEM subject (e.g., languages, history), which are not listed here. In the present study, only STEM subjects within physical/natural sciences were investigated (e.g., medical subjects were excluded).

### Measures

A questionnaire was developed by the six partners of the SESTEM consortium. They contributed according to their respective field of expertise and negotiated the specific constructs of the questionnaire, measurement approaches, and scoring systems. This kind of expert negotiation was chosen to ensure the validity of the questionnaire as well as to meet the different goals of the project. This negotiation paid attention to checks and balances to gather as much information as necessary and to keep the questionnaire as short as possible. During this process, an English version of the questionnaire was developed and then translated into five other languages including German. All questionnaire versions were combined to a multi-language questionnaire in LimeSurvey. Thus, students were able to choose their preferred language at the start of the online questionnaire. This online-survey included questions about:

1. Students’ *majors* or study subjects. Students could name up to three study subjects that were part of their degree. These were classified according to their respective proportion of women based on the German first-year students’ statistics [Destatis (Statistisches Bundesamt), 2013].
2. *Parents’ professions*. The professions were entered as text and later classified as a STEM or a non-STEM field.
3. *Intrinsic* (five items) and *extrinsic motivations* (two items, see examples of items in Table 1). Higher values, measured on a five-point Likert scale, indicate a higher level of motivation.
4. A*cademic self-concept in STEM* was measured by four items that applied a five-point Likert scale (see Table 1). Higher values indicate a more positive self-concept.
5. *School factors*. These had two aspects: the first related to students’ favorite subjects in STEM. For that, students were asked about their three most favorite subjects in school as free text. The answers were coded as STEM/non-STEM, and the STEM subjects were summed up to a score. For the analyses in this manuscript, this score only includes STEM subjects that are considered as a “male” domain (excluding, e.g., biology). Higher values indicate a higher number of favorite STEM subjects. The second aspect of the school factors is related to school support for STEM. Students were asked three questions concerning specific school teachers or activities that encouraged students’ interest in STEM (e.g., “Were there specific school activities based around STEM, such as school visits or special projects?”). Positive answers were summed up to a score and by multiplication adjusted to a range between 0 (no activities) and 5 (all activities).
6. *Family factors.* They describe support students received by family and peers (e.g., parents, siblings, friends). Students were asked whether they received support for homework or for career decisions in different subjects (e.g., math, science) and from whom they received support (e.g., “Who helped you with your science homework? [Brother or sister]”). Positive answers were summed up to a score for the subject and for the person giving support and by multiplication adjusted to a range between 0 (no support) and 1 (full support). This manuscript applies three variables for the analyses: support for math, support for science, and support by parents.

**Table 1:** Overview of the scales used for the study with the number of items, an example, and the internal consistency.

Scale	Items	Example	Cronbach’s *α*
Academic self-concept STEM	4	“I am not skilled enough in mathematics for choosing a career in STEM”	0.82
Intrinsic motivation	5	“I want to work in STEM to contribute to scientific and technical developments”	0.71
Extrinsic motivation	2	“The high salaries make a career in STEM attractive to me”	0.73

Note: The Cronbach’s *α* calculated for the whole German sample (567 students). This larger sample included students with majors, which did not qualify for inclusion in the present study. According to Paechter et al. (2013), Cronbach’s *α* with 0.70 and more can be considered a satisfying indicator of the internal consistency of a scale. If necessary, values for academic self-concepts were recoded, so that higher values indicate a higher self-concept.

Table 1 shows the characteristics of the Likert scales including the number of items, an exemplary item, and the internal consistency of the scale. Missing items of singles scales were imputed; missing scales were treated as missing. We used the values of the skewness and kurtosis to analyze the distribution of the data. West et al. (1995) set the criteria for indicators used in structural equation models at a value of >2 for skewness and >7 for kurtosis for deviation from normal distribution. All scales meet the criteria of normal distribution.

## Results

### Descriptive Statistics for the STEM-LPF Sample

Of the 284 students in STEM-LPF, 50.4% of the students (134) had a father and 11.3% (30) had a mother working in a STEM profession. Most students showed a very positive STEM self-concept (*M* = 4.58; the means described in the following related to a scale of 1–5, with 1 as the lowest value and 5 as the highest value). Intrinsic (*M* = 3.99) and extrinsic motivations (*M* = 3.81) were positive. With respect to school factors, 50 students had three favorite STEM subjects at school, 141 students had two, 84 had just one, while 9 had favorite non-STEM subjects (*M* = 1.82). They received a moderate amount of STEM support in school (*M* = 2.33 of a maximum of 5). Considering family factors, the amount of parents’ support in math (*M* = 0.14 of a maximum of 1) and STEM (*M* = 0.14) was low. General support by the parents was low to medium (*M* = 0.36). Table 2 provides an overview of all scales, including their value range, their means, and their standard deviations.

**Table 2:** Ranges, means, standard deviations, and *n* for the reported scales, students in the STEM-LPF sample.

	Range	*M*	*SD*	*n*
Motivation				
*Intrinsic*	1 … 5	3.99	0.54	284
*Extrinsic*	1 … 5	3.81	0.74	284
Academic self-concept STEM	1 … 5	4.58	0.55	284
School factors				
*STEM favorites*	0 … 3	1.82	0.75	277
*School support*	0 … 5	2.33	2.07	277
Family factors				
*Mathematics support*	0 … 1	0.14	0.19	284
*STEM support*	0 … 1	0.14	0.19	284
*Parent general support*	0 … 1	0.36	0.19	284

Table 3 provides an overview of the bivariate correlations between the variables.

**Table 3:** Bivariate correlations between variables in the STEM-LPF sample.

	(2)	(3)	(4)	(5)	(6)	(7)	(8)
Intrinsic motivation (1)	0.327**	0.325**	0.103	−0.057	0.079	−0.029	0.077
Extrinsic motivation (2)	1.000	0.159**	0.110	−0.015	0.008	0.031	0.067
Academic self-concept STEM (3)		1.000	0.094	−0.091	−0.087	−0.061	−0.003
STEM favorites (4)			1.000	−0.210**	−0.034	−0.020	0.007
School support (5)				1.000	0.028	0.068	0.004
Mathematics support (6)					1.000	0.604**	0.618**
STEM support (7)						1.000	0.621**
Parent general support (8)							1.000

Note: Significant correlations are marked with asterisks (**p < 0.01).

### Descriptive Statistics for the STEM-MPF Sample

Of the 185 students in STEM-MPF, 53.1% of the students (93) had a father and 8.6% (15) had a mother in a STEM profession. Most students showed a very positive STEM self-concept (*M* = 4.20; the means described in the following related to a scale of 1–5, with 1 as the lowest value and 5 as the highest value). Intrinsic (*M* = 3.79) and extrinsic motivations (*M* = 3.40) were positive. With respect to school factors, 22 students had three favorite STEM subjects at school, 88 students had two, 66 had just one, while 9 had favorite non-STEM subjects (*M* = 1.66). They received a moderate amount of STEM support in school (*M* = 2.38 of a maximum of 5). Considering family factors, the amount of parents’ support in math (*M* = 0.20 of a maximum of 1) and STEM (*M* = 0.15) was low. General support by the parents was low to medium (*M* = 0.38). Table 4 provides an overview of all scales, including their value range, their means, and their standard deviations.

**Table 4:** Ranges, means, standard deviations, and *n* for the reported scales, students in the STEM-MPF sample.

	Range	*M*	SD	*n*
Motivation				
*Intrinsic*	1 … 5	3.79	0.67	185
*Extrinsic*	1 … 5	3.40	0.86	185
Academic self-concept STEM	1 … 5	4.20	0.70	185
School factors				
*STEM favorites*	0 … 3	1.66	0.75	174
*School support*	0 … 5	2.38	2.12	174
Family factors				
*Mathematics support*	0 … 1	0.20	0.21	185
*STEM support*	0 … 1	0.15	0.19	185
*Parent general support*	0 … 1	0.38	0.21	185

Table 5 provides an overview of the bivariate correlations between the variables.

**Table 5:** Bivariate correlations between variables in the STEM-MPF sample.

	(2)	(3)	(4)	(5)	(6)	(7)	(8)
Intrinsic motivation (1)	0.410**	0.380**	0.259**	−0.201**	0.022	−0.011	0.077
Extrinsic motivation (2)	1.000	0.299**	0.230**	−0.284**	−0.117	−0.140	−0.050
Academic self-concept STEM (3)		1.000	0.395**	−0.195*	−0.150*	−0.092	0.023
STEM favorites (4)			1.000	−0.251**	−0.146*	−0.162*	−0.089
School support (5)				1.000	0.039	0.173*	0.080
Mathematics support (6)					1.000	0.486**	0.645**
STEM support (7)						1.000	0.648**
Parent general support (8)							1.000

Note: Significant correlations are marked with asterisks (*p < 0.05, **p < 0.01).

### Research Question 1: Differences Between the STEM-LPF and the STEM-MPF Students

A multivariate analysis of variance (MANOVA) with the two groups (STEM-LPF and STEM-MPF) as independent variables and intrinsic and extrinsic motivations as dependent variables was carried out. MANOVA showed an overall significant result, *F*(2,463) = 15.96, *p* < 0.01, *η*^2^ = 0.06. Women in STEM-LPF scored significantly higher than women in STEM-MPF on intrinsic motivation, *F*(1,464) = 29.09, *p* < 0.01, *η*^2^ = 0.06, as well as on extrinsic motivation, *F*(1,464) = 12.71, *p* < 0.01, *η*^2^ = 0.03.

A *t*-test between both groups with the academic STEM self-concept as dependent variable also found higher values for women in STEM-LPF, *t*(109) = 6.25, *p* < 0.01, Cohen’s *d* = 0.7.

MANOVA with the two groups (STEM-LPF and STEM-MPF) as independent variables and the school factors as dependent variables was not significant, *F*(2,448) = 2.04, *p* > 0.05, *η*^2^ = 0.01.

MANOVA with the two groups (STEM-LPF and STEM-MPF) as independent variables and family variables as dependent variables was significant, *F*(3,465) = 4.68, *p* < 0.05, *η*^2^ = 0.03. Women in STEM-MPF scored significantly higher than women in STEM-LPF on perceived parental support in math, *F*(1,467) = 11.78, *p* < 0.01, *η*^2^ = 0.02. There were significant differences neither for perceived parental support in STEM, *F*(1,467) = 0.34, *p* > 0.05, *η*^2^ = 0.00, nor for general parental support, *F*(1,467) = 1.43, *p* > 0.05, *η*^2^ = 0.00.

### Research Question 2a: Latent Regression Analysis for STEM-LPF Students

Latent regression analysis was used to test the relationships between the variables in a multivariate, multiple regression context. Structural relationships between multiple dependent variables and multiple independent variables can be analyzed simultaneously. Regression analyses are specified at the latent level and are corrected for measurement error at the level of the independent and dependent variables. Latent regression analyses have the advantage that the relationships between variables in the regression model can be estimated more accurately (Geiser, 2013). The data were analyzed with Mplus 6 using a maximum likelihood estimator. The goodness of fit of the data to the hypothesized model was assessed using the following indices: *χ*^2^/df, comparative fit index (CFI), root mean square error of approximation (RMSEA), and standardized root mean square residual (SRMR). In general, values of *χ*^2^/df < 2, CFI > 0.95, RMSEA < 0.05, and SRMR < 0.05 are considered indicators of good model fit. The model fit indices suggest a good fit of the latent regression analysis model for STEM-LPF: *χ*^2^/df = 1.039; CFI = 0.999; RMSEA = 0.012; SRMR = 0.022.

Table 6 displays the standardized solutions for the latent regression analysis with the school and family factors. Each factor comprises different variables. The model shows that the two indicators STEM favorites at school (*β* = 0.593) and school support (*β* = −0.355) are related to the latent school factor. The three indicators support in mathematics (*β* = 0.778), support in STEM (*β* = 0.777), and support by parents (*β* = 0.797) are related to the latent factor family.

**Table 6:** Standardized coefficients for the latent regression analysis for the STEM-LPF sample.

Observed variable	Latent factor	*β*	SE	*p*
STEM favorites	*School*	0.593	0.250	0.018
Support in school		−0.355	0.157	0.024
Mathematics support	*Family*	0.778	0.034	<0.001
Science support		0.777	0.034	<0.001
Parent support		0.797	0.034	<0.001

**Figure 1:**
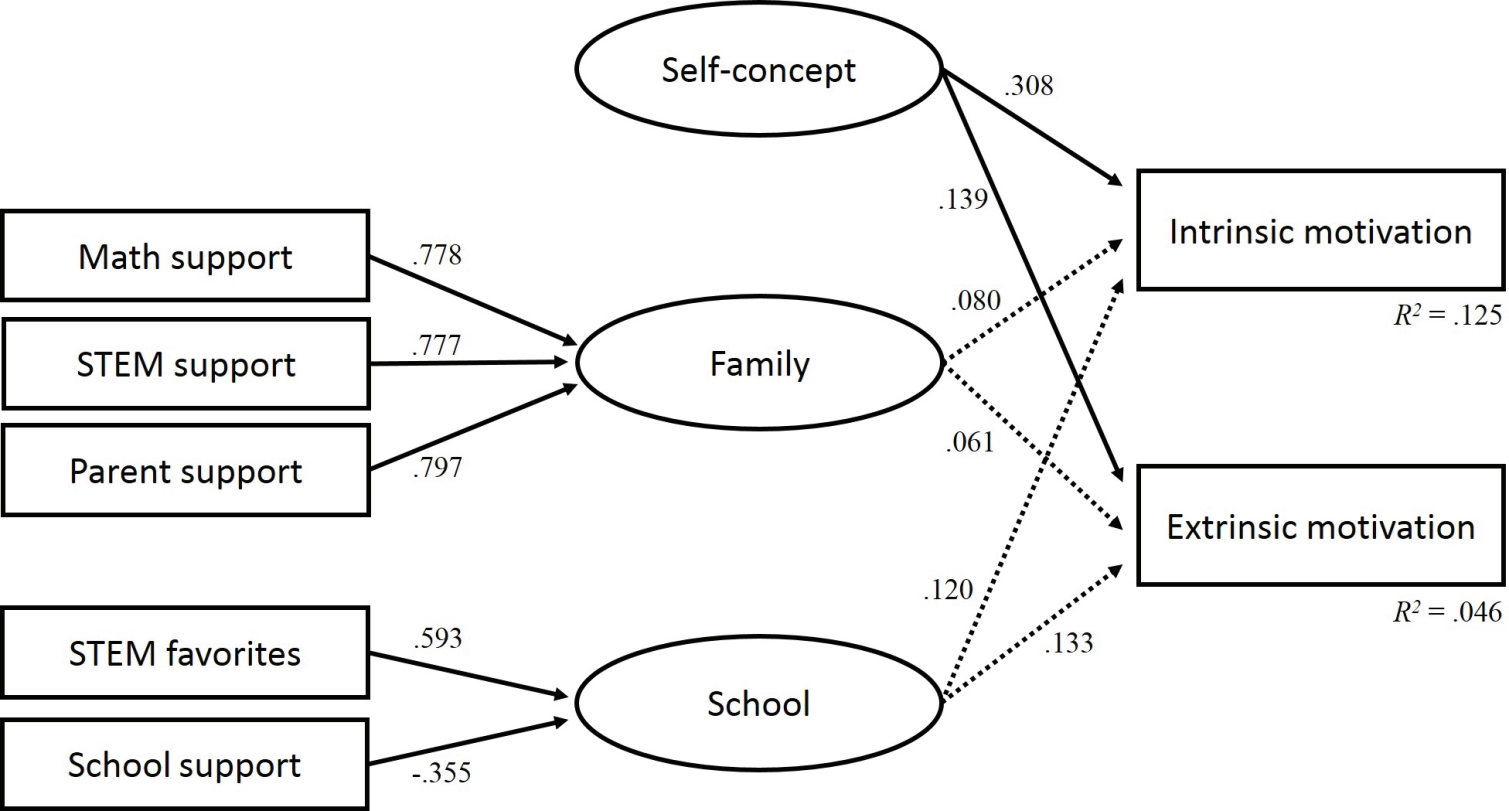
Latent regression analysis for the STEM-LPF sample.

The regression coefficients between the school and family factors and the self-concept as predictor variables and intrinsic and extrinsic motivations as criterion variables show the following results: students in STEM-LPF with higher levels of academic self-concept in STEM report higher intrinsic (*β* = 0.308) and extrinsic motivations (*β* = 0.139). There were no significant correlations between the school and family factors and intrinsic and extrinsic motivations. The total variance of intrinsic and extrinsic motivations that can be explained is *R*^2^ (intrinsic) = 0.125 and *R*^2^ (extrinsic) = 0.046. Figure 1 gives an overview of the indicators and factors of the latent regression analysis model.

### Research Question 2b: Latent Regression Analysis for Students in Subjects With a Moderate Proportion of Females

The model fit indices suggest a good fit of the latent regression analysis model for STEM-MPF: *χ*^2^/df = 1.759; CFI = 0.970; RMSEA = 0.064; SRMR = 0.040. Table 7 displays the standardized solutions for the latent regression analysis with the school and family factors. The model shows that the two indicators STEM favorites at school (*β* = 0.591) and school support (*β* = −0.431) are related to the latent school factor. The three indicators support in mathematics (*β* = 0.705), support in STEM (*β* = 0.710), and support by parents (*β* = 0.910) are high, positively related to the latent family factor.

**Table 7:** Standardized coefficients for the latent regression analysis for the students in subjects with a moderate proportion of females.

Observed variable	Latent factor	*β*	SE	*p*
STEM favorites	*School*	0.591	0.100	<0.001
Support in school		−0.431	0.090	<0.001
Mathematics support	*Family*	0.705	0.047	<0.001
Science support		0.710	0.048	<0.001
Parent support		0.910	0.041	<0.001

**Figure 2:**
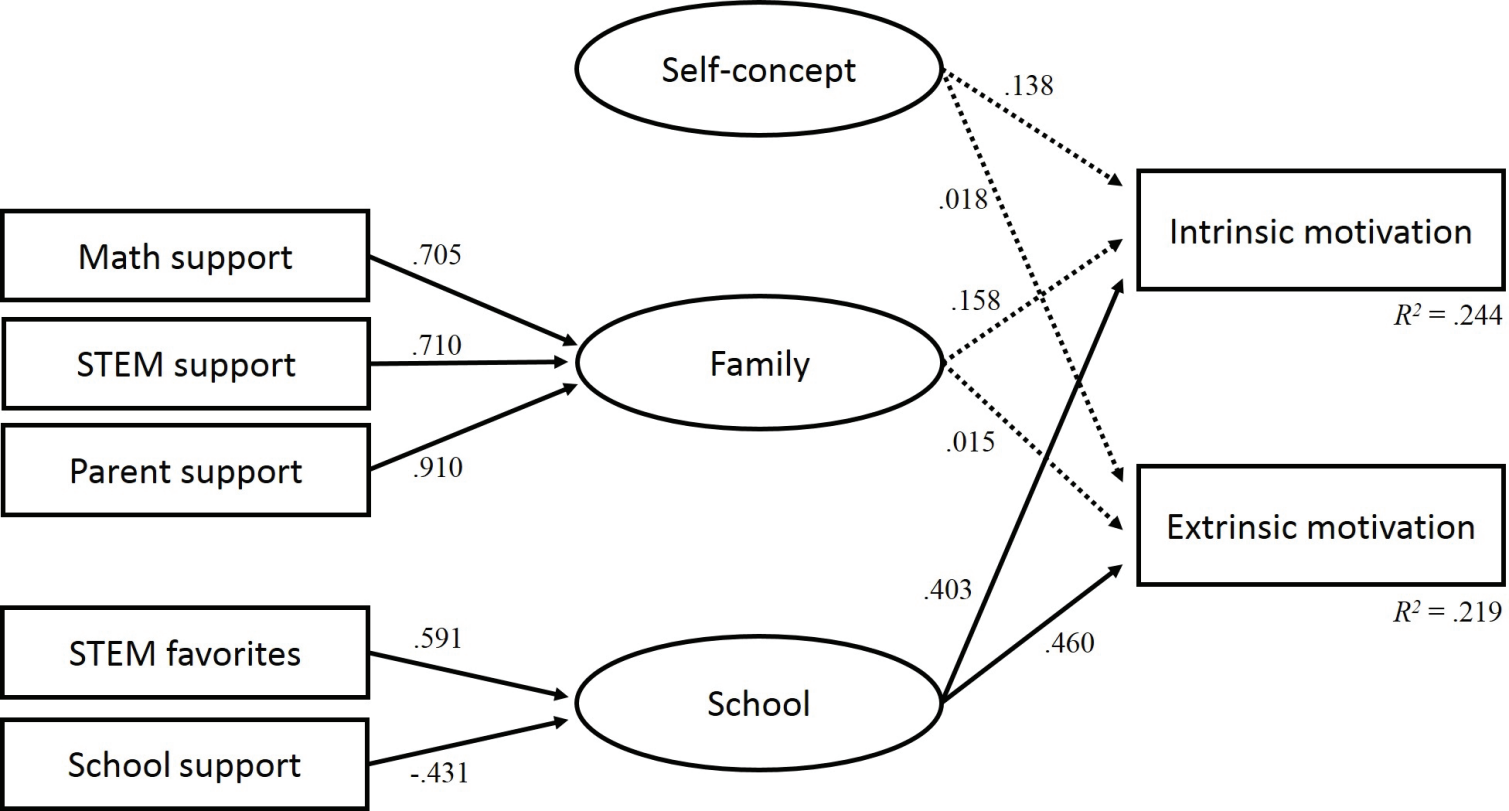
Latent regression analysis for the students in subjects with a moderate proportion of females.

The regression coefficients between the school and family factors and the STEM self-concept as predictor variables and intrinsic and extrinsic motivations as criterion variables show the following results: the model shows a moderate relationship between the latent school factor and students’ intrinsic (*β* = 0.403) and extrinsic (*β* = 0.461) motivations. Students in STEM-MPF who reported a higher number of favorite STEM subjects in school have higher intrinsic and extrinsic motivations in STEM. By contrast, higher levels of school support indicate lower intrinsic and extrinsic motivations in STEM. There were no significant correlations between academic self-concept in STEM and support by the family. The total variance of intrinsic and extrinsic motivations that can be explained by the factors is *R*^2^ (intrinsic) = 0.244 and *R*^2^ (extrinsic) = 0.299. Figure 2 gives an overview of the indicators and factors of the latent regression analysis model.

## Discussion

Consistent with the suggestions to differentiate between STEM domains (Watt et al., 2017), this study classified STEM subjects into two groups according to their proportion of female students. Distinct differences and patterns could be found for the two groups.

### STEM Self-Concept as a Key Predictor for Motivation in STEM-LPF Subjects

Women in STEM subjects with a low proportion of females showed a significantly higher STEM self-concept and significantly higher intrinsic and extrinsic motivations than women in STEM-MPF. The higher STEM self-concept might be a result of a selection process in which only the most confident and able females decide on a career in strongly male-dominated fields.

Only the STEM self-concept contributed significantly to intrinsic and extrinsic motivations in the latent regression analysis. Neither family factors nor school factors received significant *β*-weights. Generally, research points at a strong influence of the STEM self-concept on the motivation to achieve in a subject and invest effort into it (Guay et al., 2010). Both self-determination theory and research on the academic self-concept (Marsh and Craven, 2005; Guay et al., 2010) assume that perceiving oneself as able and competent increases intrinsic motivation and achievement. In the present study, this kind of high self-concept in STEM was related to facets of intrinsic motivation, for example, the wish to contribute positively to scientific and technical developments in the respective field of study (see item example in Table 1).

STEM self-concept was also significantly related to extrinsic motivation. However, in comparison to intrinsic motivation, the *β*-weight was much smaller. This result might be explained by the women’s previous experiences. A high self-concept and intrinsic motivation are optimal requirements for long-lasting, persistent involvement in a domain. By contrast, involvement based on external motivation is much more liable to break down in case of failure, disappointment, stereotypes, etc. (Guay et al., 2010) – all obstacles which women in extremely male-dominated subjects are likely to encounter. As a result, an especially strong link between intrinsic motivation and self-concept can be expected.

### School Factors as Key Predictors for Motivation in STEM-MPF Subjects

Only school factors contributed to intrinsic and extrinsic motivations in the latent regression analysis. Neither family factors nor the academic self-concept received significant *β*-weights.

Former experiences and preferences expressed as STEM subjects being favorite subjects in school contributed positively to intrinsic and extrinsic motivations. Favorite subjects can be seen as indicators of intrinsic motivation. So in this case, former appraisal of STEM (to be sure, assessed in hindsight) contributes to present intrinsic motivation. STEM subjects as favorite subjects also contribute to extrinsic motivation, e.g., expectations of a good salary, etc.

By contrast (and at a first glance, somewhat counterintuitively), the variable of school support contributed negatively to intrinsic and extrinsic motivations. It expresses assessments of the support provided by teachers and the school as such, e.g., special activities that encouraged interest in STEM. There are different explanations for this result. It might be that, in hindsight, these activities in school have little to do with present academic experiences and demands. This variable would therefore tend to impede current motivation in STEM. Another reason might be that these kinds of school activities were perceived as intrusive, perhaps carrying the message that STEM subjects are not interesting *per se*, but need special encouragement and emphasis in order to be regarded as attractive, especially for girls. It speaks for this assumption that the variables STEM favorites and school support correlate negatively. Bhanot and Jovanovic (2005) showed for mathematics that parental support often carries these kinds of “hidden” messages. When this is the case, special activities and encouragement provided by teachers might backfire and discourage rather than encourage motivation and interest.

### Differences Between Women in STEM-LPF and STEM-MPF Subjects

Differences between the two groups of women concern mainly the academic STEM self-concept and intrinsic and extrinsic motivations. Women in STEM subjects with a low proportion of females excelled by a higher STEM self-concept. One may assume that only women with a strong STEM self-concept that “inoculates” against the multiple barriers (stereotypes, lack of support systems in the family or by peers; Dasgupta and Stout, 2014; Wang et al., 2015) will lastly decide for and stick to a career in a STEM-LPF field. As self-concept is formed in early years of childhood and becomes more stable over time (Nagy et al., 2010; Ertl et al., 2017), this result points at the importance to build up a strong STEM self-concept already in early school years.

However, the decision to take up a study in a STEM-LPF field needs not only a strong self-concept but also a high degree of persistence and stamina. Therefore, it is not surprising that the difference between women in STEM-LPF and STEM-MPF concerning their STEM self-concept is accompanied by a higher degree of intrinsic and extrinsic motivations. Students with higher motivation in a subject invest more time and effort in learning and performance and thus meet important requirements for academic success. In such a long-term context, intrinsic motivation is especially important (Van Soom and Donche, 2014; Luttenberger et al., 2018).

Of the family and school factors, only one variable distinguished between the two groups. Women in STEM-LPF perceived lower math support from the family than women in STEM-MPF. In the light of differences in self-concept and motivation, it seems probable that these women did not perceive their parents’ support as so important for pursuing their STEM career as they already had strong internal personal motives for their decision.

## Limitations and Strengths of the Study

A strength of this study is its relatively large sample with 469 individuals of a “rare species” – women in STEM subjects, with a large number of them even studying subjects with a low proportion of females.

This study’s limitations may perhaps be found in its methodology that uses a cross-sectional design instead of a longitudinal design. This has implications for drawing inferences and causalities. Thus, it is not possible to investigate whether relationships, for example, between school and family factors and motivational variables, are mediated by the STEM self-concept. To investigate such causalities, a longitudinal design would be desirable. However, achieving a sample size like the one in the present study with a longitudinal design that covers ideally primary to secondary and to tertiary education is nearly impossible. Nevertheless, the study gives new insights into the motivation of women in university studies that are connoted as typically male domains. An important point of the survey is that it does not consider these studies as a homogeneous domain but takes a differentiated view.

## Ethics Statement

This study was performed in accordance with the 1964 Declaration of Helsinki and the American Psychological Association’s Ethics Code. In accordance with the national and institutional requirements, review and approval were not required for this study. Participants gave consent to participate in the study by submitting the online questionnaire.

## Author Contributions

SL, MP, and BE have made a substantial, direct and intellectual contribution to the work, and approved it for publication.

### Conflict of Interest Statement

The authors declare that the research was conducted in the absence of any commercial or financial relationships that could be construed as a potential conflict of interest.

## References

[ref1] Adya M. Kaiser K. M. (2005). Early determinants of women in the IT workforce: a model of girls’ career choices. Inf. Technol. People 18, 230–259. 10.1108/09593840510615860

[ref2] Aeschlimann B. Herzog W. Makarova E. (2016). How to foster students’ motivation in mathematics and science classes and promote students’ STEM career choice. A study in Swiss high schools. Int. J. Educ. Res. 79, 31–41. 10.1016/j.ijer.2016.06.004

[ref3] Altermatt E. R. Pomerantz E. M. Ruble D. N. Frey K. S. Greulich F. K. (2002). Predicting changes in children’s self-perceptions of academic competence: a naturalistic examination of evaluative discourse among classmates. Dev. Psychol. 38, 903–917. 10.1037/0012-1649.38.6.903, PMID: 12428703

[ref4] Bhanot R. Jovanovic J. (2005). Do parents’ academic gender stereotypes influence whether they intrude on their children’s homework? Sex Roles 52, 597–607. 10.1007/s11199-005-3728-4

[ref5] Blickenstaff J. (2005). Women and science careers: leaky pipeline or gender filter? Gend. Educ. 17, 369–386. 10.1080/09540250500145072

[ref6] Buchmann M. Kriesi I. Pfeifer A. Sacchi S. (2002). Halb drinnen – halb draussen. Analysen zur Arbeitsmarktintegration von Frauen in der Schweiz. [Half inside – half outside. Analyses on the integration of women into the labor market in Switzerland.]. (Zürich: Rüegger).

[ref7] Bussek K. Bandura A. (1999). Social cognitive theory of gender development and differentiation. Psychol. Rev. 106, 676–713. 10.1037/0033-295X.106.4.676, PMID: 10560326

[ref8] Center of Excellence Women and Science (2014). Studentinnenanteil in Mathematik/Naturwissenschaften und Ingenieurwissenschaften (ISCED 5-6) im internationalen Vergleich (2011). [Proportion of female students in mathematics/sciences and engineering (ISCED 5-6) in an international comparison]. Retrieved from: http://www.gesis.org/cews/fileadmin/cews/www/statistiken/08_d.gif (Accessed April 02, 2014).

[ref9] Dasgupta N. Stout J. G. (2014). Girls and women in science, technology, engineering and mathematics: STEMing the tide and broadening participation in STEM careers. Policy Insights Behav. Brain Sci. 1, 21–29. 10.1177/2372732214549471

[ref10] Deci E. L. Ryan R. M. (1991). “A motivational approach to self: Integration in personality” in Nebraska symposium on motivation: Perspectives on motivation. ed. Dienstbier R., vol. 38 (Lincoln, NE: University of Nebraska Press), 237–288. 2130258

[ref12] Destatis (Statistisches Bundesamt) (2013). Bildung und kultur. Studierende an hochschulen. Wintersemester 2012/2013. [Education and culture. Students at universities. Winter term 2012/2013] (Wiesbaden: Statistisches Bundesamt).

[ref13] Dick T. P. Rallis S. F. (1991). Factors and influences on high school students’ career choices. J. Res. Math. Educ. 22, 281–292. 10.2307/749273

[ref14] Dickhäuser O. Meyer W.-U. (2006). Gender differences in young children’s math ability attributions. Psychol. Sci. 48, 3–16.

[ref15] Dresel M. Schober B. Ziegler A. (2007). “Golem und Pygmalion. Scheitert die Chancengleichheit von Mädchen im mathematisch-naturwissenschaftlich-technischen Bereich am geschlechtsstereotypen Denken der Eltern? [Golem and Pygmalion. Do equal opportunities for girls in mathematical-scientific-technical domains fail because of their parents’ gender-stereotype thinking?]” in Erwartungen in himmelblau und rosarot. Effekte, Determinanten und Konsequenzen von Geschlechterdifferenzen in der Schule. eds. Ludwig P. H. Ludwig H. (Weinheim: Juventa), 61–81.

[ref16] Ellis J. Fosdick B. K. Rasmussen C. (2016). Women 1.5 times more likely to leave STEM pipeline after calculus compared to men: lack of mathematical confidence a potential culprit. PLoS One 11:e0157447. 10.1371/journal.pone.0157447, PMID: 27410262 PMC4943602

[ref17] Else-Quest N. M. Hyde J. S. Linn M. C. (2010). Cross-national patterns of gender differences in mathematics: a meta-analysis. Psychol. Bull. 136, 103–127. 10.1037/a0018053, PMID: 20063928

[ref18] Ertl B. Luttenberger S. Paechter M. (2014). Stereotype als Einflussfaktoren auf die Motivation und die Einschätzung der eigenen Fähigkeiten bei Studentinnen in MINT-Fächern. [Stereotypes as influencing factors on motivation and assessment of one’s own skills of female students in STEM-subjects]. Gruppendynamik und Organisationsberatung 45, 419–440. 10.1007/s11612-014-0261-3

[ref19] Ertl B. Luttenberger S. Paechter M. (2017). The impact of gender stereotypes on the self-concept of female students in STEM subjects with an under-representation of females. Front. Psychol. 8:703. 10.3389/fpsyg.2017.00703, PMID: 28567022 PMC5434750

[ref20] Frenzel A. C. Goetz T. Pekrun R. Watt H. M. G. (2010). Development of mathematics interest in adolescence: influences of gender, family, and school context. J. Res. Adolesc. 20, 507–537. 10.1111/j.1532-7795.2010.00645.x

[ref21] Geiser C. (2013). Data analysis with mplus. (New York, NY: The Guilford Press).

[ref22] Guay F. Chanal J. Ratelle C. F. Marsh H. W. Larose S. Boivin M. (2010). Intrinsic, identified, and controlled types of motivation for school subjects in young elementary school children. Br. J. Educ. Psychol. 80, 711–735. 10.1348/000709910X499084, PMID: 20447334

[ref23] Gunderson E. A. Ramirez G. Levine S. C. Beilock S. L. (2012). The role of parents and teachers in the development of gender-related math attitudes. Sex Roles 66, 153–166. 10.1007/s11199-011-9996-2

[ref24] Hoferichter F. Lätsch A. Lazarides R. Raufelder D. (2018). The big-fish-little-pond effect on the four facets of academic self-concept. Front. Psychol. 9:1247. 10.3389/fpsyg.2018.01247, PMID: 30079044 PMC6062938

[ref25] Ihsen S. Höhle E. A. Baldin D. (2013). “Spurensuche!: Entscheidungskriterien für Natur-bzw. Ingenieurwissenschaften und mögliche Ursachen für frühe Studienabbrüche von Frauen und Männern an TU9-Universitäten. [Tracking!: decision criteria for science and engineering and possible causes for early dropouts of women and men at TU9 universities.]” in TUM gender- und diversity-studies, vol. 1 (Berlin: LIT).

[ref26] Jacobs J. E. Lanza S. Osgood D. W. Eccles J. S. Wigfield A. (2002). Changes in children’s self-competence and values: Gender and domain differences across grades one through twelve. Child Dev. 73, 509–527. 10.1111/1467-8624.00421, PMID: 11949906

[ref27] Kessels U. (2015). Bridging the gap by enhancing the fit: how stereotypes about STEM clash with stereotypes about girls. Int. J. Gender, Sci. Technol. 7, 280–296.

[ref28] Lazarides R. Ittel A. (2012). Instructional quality and attitudes toward mathematics: do self-concept and interest differ across students’ patterns of perceived instructional quality in mathematics classrooms? Child Dev. Res. 2012. 10.1155/2012/813920

[ref500] Luttenberger S. Wimmer S. Paechter M. (2018). Spotlight on math anxiety. Psychol. Res. Behav. Manag. 11, 311–322. 10.2147/PRBM.S141421 30123014 PMC6087017

[ref29] Macher D. Paechter M. Papousek I. Ruggeri K. Freudenthaler H. H. Arendasy M. (2013). Statistics anxiety, state anxiety during an examination, and academic achievement. Br. J. Educ. Psychol. 83, 535–549. 10.1111/j.2044-8279.2012.02081.x, PMID: 24175681

[ref30] Marsh H. W. Craven R. G. (2005). “A reciprocal effects model of the causal ordering of self-concept and achievement: new support for the benefits of enhancing self-concept” in New frontiers for self research. eds. Marsh H. W. Craven R. G. McInerney D., vol. 2 (Greenwich, CT: Information Age), 15–52.

[ref31] Marsh H. W. Scalas L. F. (2011). “Self-concept in learning: reciprocal effects model between academic self-concept and academic achievement” in Social and emotional aspects of learning. ed. Järvela S. (Amsterdam: Elsevier), 191–197.

[ref32] Murphy M. C. Steele C. M. Gross J. J. (2007). Signaling threat: how situational cues affect women in math, science, and engineering settings. Psychol. Sci. 18, 879–885. 10.1111/j.1467-9280.2007.01995.x 17894605

[ref33] Nagy G. Watt H. M. G. Eccles J. S. Trautwein U. Lüdtke O. Baumert J. (2010). The development of students’ mathematics self-concept in relation to gender: different countries, different trajectories? J. Res. Adolesc. 20, 482–506. 10.1111/j.1532-7795.2010.00644.x

[ref34] Nosek B. A. Smyth F. L. (2011). Implicit social cognitions predict sex differences in math engagement and achievement. Am. Educ. Res. J. 48, 1125–1156. 10.3102/0002831211410683

[ref35] OECD (2015). The ABC of gender equality in education: Aptitude, behaviour, confidence. (Paris: PISA, OECD Publishing).

[ref36] Paechter M. Rebmann K. Schlömer T. Mokwinski B. Hanekamp Y. Arendasy M. (2013). Development of the Oldenburg Epistemic Beliefs Questionnaire (OLEQ), a German questionnaire based on the Epistemic Belief Inventory (EBI). Curr. Issues Educ. 16, 1–18. Retrieved from http://cie.asu.edu/ojs/index.php/cieatasu/article/view/1035

[ref37] Parsons J. E. Adler T. Meece J. L. (1984). Sex differences in achievement: a test of alternate theories. J. Pers. Soc. Psychol. 46, 26–43. 10.1037/0022-3514.46.1.26

[ref38] Robnett R. D. Leaper C. (2012). Friendship groups, personal motivation, and gender in relation to high school students’ STEM career interest. J. Res. Adolesc. 23, 652–664. 10.1111/jora.12013

[ref39] Schunk D. H. Meece J. L. Pintrich P. R. (2014). Motivation in education: Theory, research, and applications. 4th edn. (Boston: Pearson Education).

[ref40] Senler B. Sungur S. (2009). Parental influences on students’ self-concept, task value beliefs, and achievement in science. Span. J. Psychol. 12, 106–117. 10.1017/S1138741600001529, PMID: 19476224

[ref41] Sonnert G. (2009). Parents who influence their children to become scientists: effects of gender and parental education. Soc. Stud. Sci. 39, 927–941. 10.1177/0306312709335843, PMID: 20506745

[ref42] Stevenson H. W. Newman R. S. (1986). Long-term prediction of achievement and attitudes in mathematics and reading. Child Dev. 57, 646–659. 10.2307/1130343, PMID: 3720396

[ref43] Tiedemann J. (2000). Parents’ gender stereotypes and teachers' beliefs as predictors of children’s concept of their mathematical ability in elementary school. J. Educ. Psychol. 92, 144–151. 10.1037/0022-0663.92.1.144

[ref44] Valentine J. C. DuBois D. L. Cooper H. (2004). The relation between self-beliefs and academic achievement: a meta-analytic review. Educ. Psychol. 39, 111–133. 10.1207/s15326985ep3902_3

[ref45] Van Soom C. Donche V. (2014). Profiling first-year students in STEM programs based on autonomous motivation and academic self-concept and relationship with academic achievement. PLoS One 9:e112489. 10.1371/journal.pone.0112489, PMID: 25390942 PMC4229203

[ref46] Vermeer H. J. Boekaerts M. Seegers G. (2000). Motivational and gender differences: sixth-grade students’ mathematical problem-solving behavior. J. Educ. Psychol. 92, 308–315. 10.1037/0022-0663.92.2.308

[ref47] Wang M.-T. Degol J. Ye F. (2015). Math achievement is important, but task values are critical, too: examining the intellectual and motivational factors leading to gender disparities in STEM careers. Front. Psychol. 6. 10.3389/fpsyg.2015.00036 PMC4330678 25741292

[ref48] Watt H. M. G. (2004). Development of adolescents’ self-perceptions, values, and task perceptions according to gender and domain in 7th through 11th grade Australian students. Child Dev. 75, 1556–1574. 10.1111/j.1467-8624.2004.00757.x, PMID: 15369531

[ref49] Watt H. M. G. Hyde J. S. Petersen J. Morris Z. A. Rozek C. S. Harackiewicz J. M. (2017). Mathematics: a critical filter for STEM-related career choices? A longitudinal examination among Australian and US adolescents. Sex Roles 77, 254–271. 10.1007/s11199-016-0711-1

[ref50] West S. G. Finch J. F. Curran P. J. (1995). “Structural equation models with nonnormal variables: problems and remedies” in Structural equation modeling: Concepts, issues, and applications. ed. Hoyle R. H. (Thousand Oaks, CA: Sage), 56–75.

